# Breast filler granuloma mistaken for implant rupture: A case report

**DOI:** 10.1097/MD.0000000000033785

**Published:** 2023-06-02

**Authors:** Yong Seon Hwang, Je Yeon Byeon, Jun Hyuk Kim, Hwan Jun Choi, Mee Hye Oh, Da Woon Lee

**Affiliations:** a Department of Plastic and Reconstructive Surgery, College of Medicine, Soonchunhyang University Cheonan Hospital, Cheonan, Korea; b Institute of Tissue Regeneration, College of Medicine, Soonchunhyang University, Cheonan, Korea; c Department of Pathology, College of Medicine, Soonchunhyang University Cheonan Hospital, Cheonan, Korea.

**Keywords:** augmentation, filler, granuloma, misconception, rupture

## Abstract

Breast augmentation is usually performed by inserting implants into the breasts. However, injectable fillers are sometimes used for the convenience of both patients and surgeons. If foreign substances, such as biomaterials, are injected into the body, complications such as inflammation, granuloma, and tissue necrosis can occur owing to foreign body reactions.

**Patient concerns:**

A 39-year-old female patient visited our hospital complaining of tenderness, redness, and swelling in both breasts. The patient had undergone bilateral breast augmentation using implants 4 years prior to current consult.

**Diagnoses:**

On magnetic resonance imaging (MRI), cystic lesions and fluid collections were observed, with findings suggesting implant rupture; hence, surgery was planned to remove both implants.

**Interventions:**

Intraoperatively, the implant was malpositioned in the upper lateral portion without rupture. Capsular contracture findings were also not prominent. A large amount of inflammatory granuloma was observed and removed in the prepectoral plane, and the implants were immediately inserted into a new subpectoral plane.

**Outcomes:**

The volume of the new implant was 175 mL, which was smaller than the previous one, as per the patient preference. Cytology of the fluid from the previous implant pocket showed no evidence of malignancy, and the granuloma was identified as inflammatory tissue caused by a foreign body reaction on biopsy. The excessive protrusion of both breasts was corrected after surgery, and the patient was satisfied with the aesthetic outcomes without any complications up to 3 months after surgery.

**Lessons:**

The use of injectable fillers for breast augmentation carries the risk of misdiagnosis, and, therefore, surgeons should always exercise caution.

## 1. Introduction

Breast augmentation, one of the most popular aesthetic surgeries, is mainly performed using implants. Silicone implants are inserted into the space above or below the pectoralis major muscle under general anesthesia. Early complications of surgery involving implants include infection and fluid collection around the implants, whereas late complications include capsular contracture, implant rupture, and silicone-induced lymph node enlargement. Of these, implant rupture is the most clinically significant complication.^[1]^ In addition to complications caused by the physical damage and deformation of breast implants, malignant diseases such as breast implant-associated anaplastic large cell lymphoma (BIA-ALCL) have recently emerged as a new disease group. The incidence of the BIA-ALCL is very low. It has been reported that it is mainly related to textured implants, and a large number of cases are related to BioCell (Allergan Inc., Irvine, CA).^[2]^ In Korea, several cases of BIA-ALCL diagnosis in women with BioCell implants have been reported since 2019.^[3]^

With increased incidence in malignancies related to breast implants, such as BIA-ALCL, some patients are reluctant to insert these silicone implants. In addition, satisfaction with surgery is subjective, depending on the surgeon experience and the patient preference, which makes the patient hesitant to undergo surgery. Most importantly, patients prefer simpler procedures because of their fear of general anesthesia. From the surgeon perspective, multiple revision surgeries are often required if postoperative results are poor. Therefore, plastic surgeons are searching for simple methods to perform breast augmentation. Consequently, breast augmentation has been performed using fillers in limited cases.^[4]^ The injection of fillers can be performed under local anesthesia. Breast augmentation using fillers does not require general anesthesia and is performed by injecting fillers into the precostal plane or breast parenchyma. Patients undergo breast augmentation not only for developmental disabilities but also for reconstruction after mastectomy or for improved self-image. Indications for breast augmentation using fillers have not been established; however, they are sometimes used to fill insufficient volumes after implants are inserted.^[5]^ Similar to breast augmentation using implants, there are several complications of filler injections, and the duration and incidence of these complications vary depending on the components of the fillers.

In this paper, we report and discuss the meaning of a case of a patient with breast filler granuloma who was initially diagnosed with bilateral ruptured implants.

## 2. Patients and methods

This retrospective case study involved a patient who visited our outpatient clinic, Department of Plastic and Reconstructive Surgery. The study protocol was approved by the Institutional Review Board (number:2022-12-057). The written informed consent was obtained from the patient for publication of this case report details. All procedures were performed in accordance with the ethical standards of the institutional and/or national research committee, and the 1964 Declaration of Helsinki and its later amendments or comparable ethical standards.

## 3. Case report

**Figure 1.**
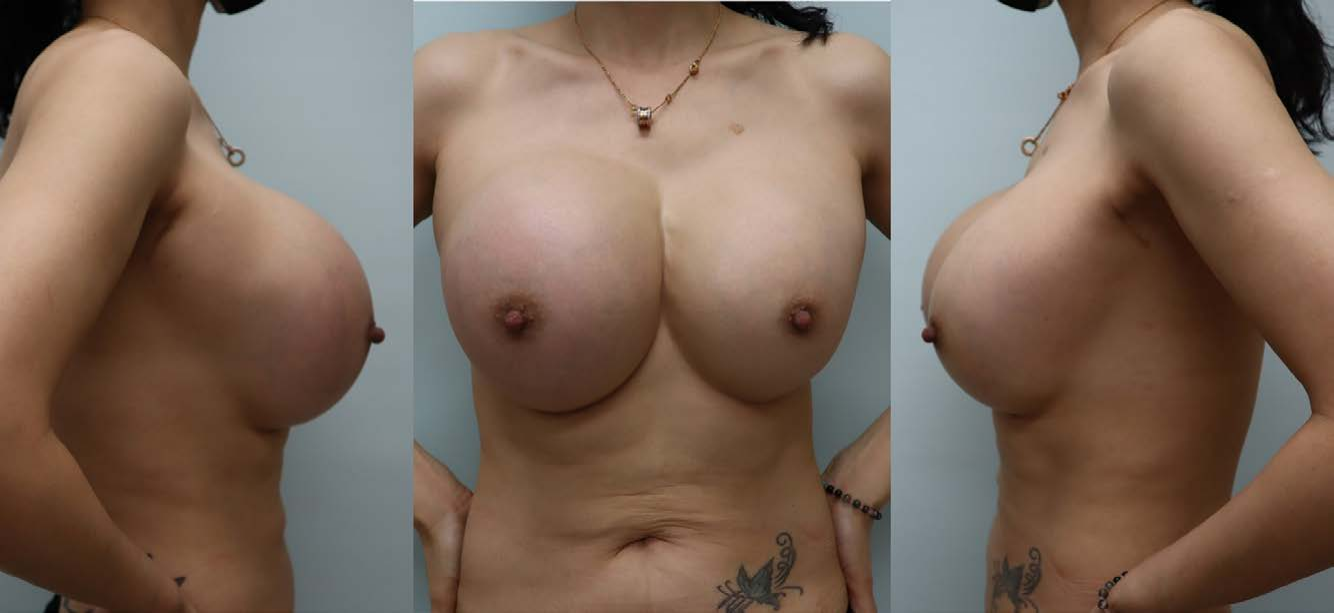
Preoperative photography. The patient complained of swelling, redness and tenderness of both breast.

A 39-year-old female patient was referred to our plastic surgery department because of redness, swelling, and tenderness in both breasts which started 6 months prior to presentation. The patient underwent augmentation mammoplasty 4 years prior at a local clinic by a non-specialist who inserted 240 cc micro-textured implants (Sebbin, France) into both breasts. An incision was made using a transaxillary approach. Three years and 6 months after surgery, the patient breasts began to swell, with the right side more swollen than the left. On physical examination, fluctuations were palpable on both sides, and symmastia was observed. Both the breasts exhibited redness, tenderness, and hardness (Fig. 1). There was no history of trauma suggestive of an implant rupture. Despite skin erythema, the patient laboratory findings (white blood cell count, C-reactive protein level, and erythrocyte sedimentation rate) were within normal ranges.

**Figure 2.**
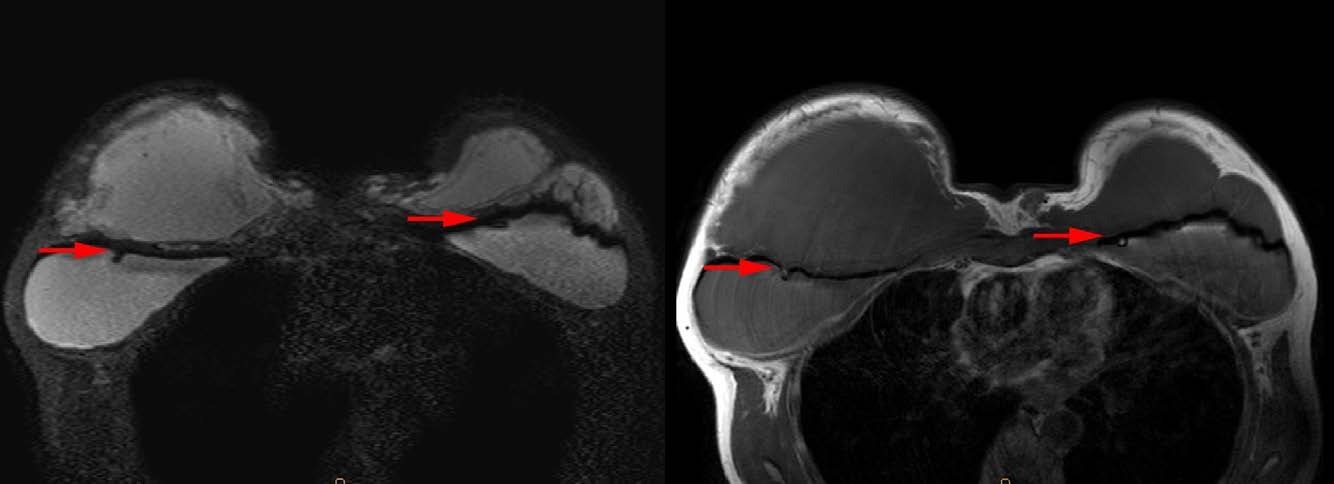
Magnetic resonance imaging. “Tear drop sign” of both implants could be seen on T1 and T2 imaging (red arrows), suggesting suspicious rupture of the implants.

Unspecified fluid collection and mass formation were suspected. Magnetic resonance imaging (MRI) revealed a massive volume of extracapsular fluid collection, making it necessary to differentiate between inflammation and malignancy, such as BIA-ALCL. In addition, intra- or extracapsular rupture was strongly suspected because the thickness of the capsule was not constant, and the internal lining and lobulated contours of both implants were observed (Fig. 2). Differential diagnoses included implant rupture, severe capsular contracture, and BIA-ALCL.

**Figure 3.**
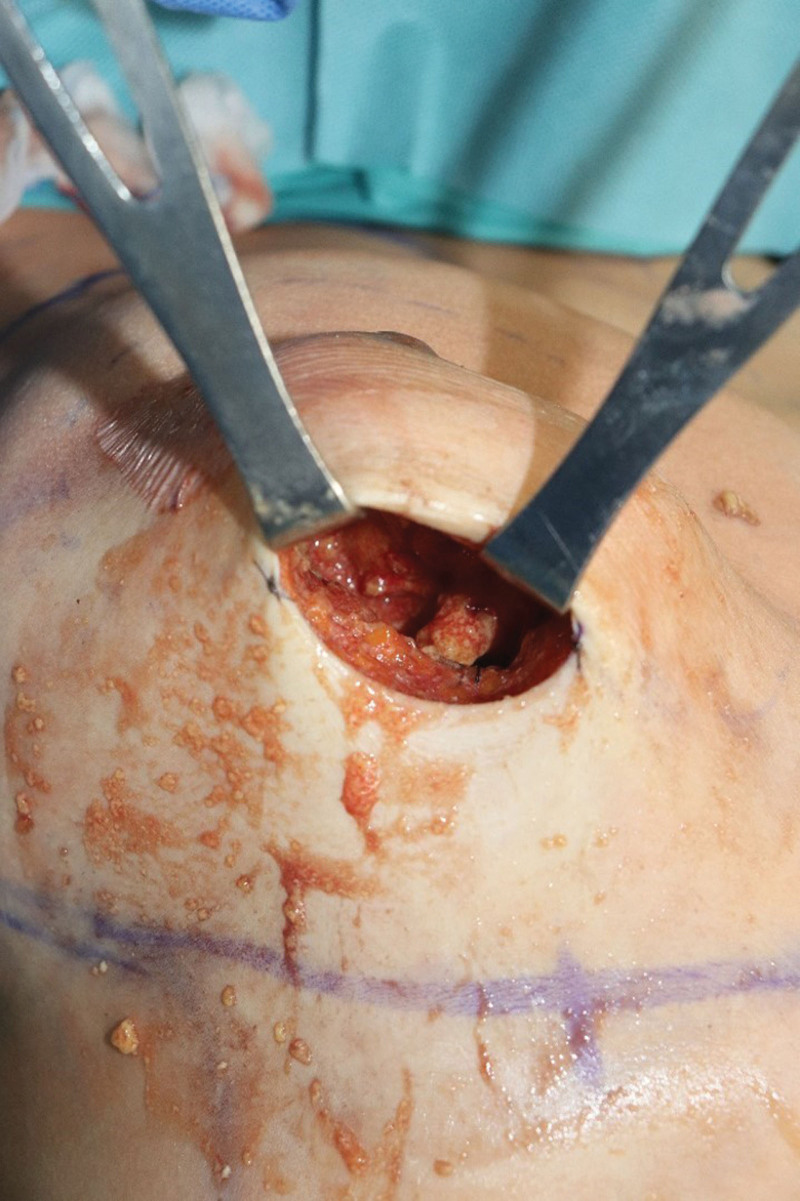
Intraoperative photography. The fat necrosis was found.

**Figure 4.**
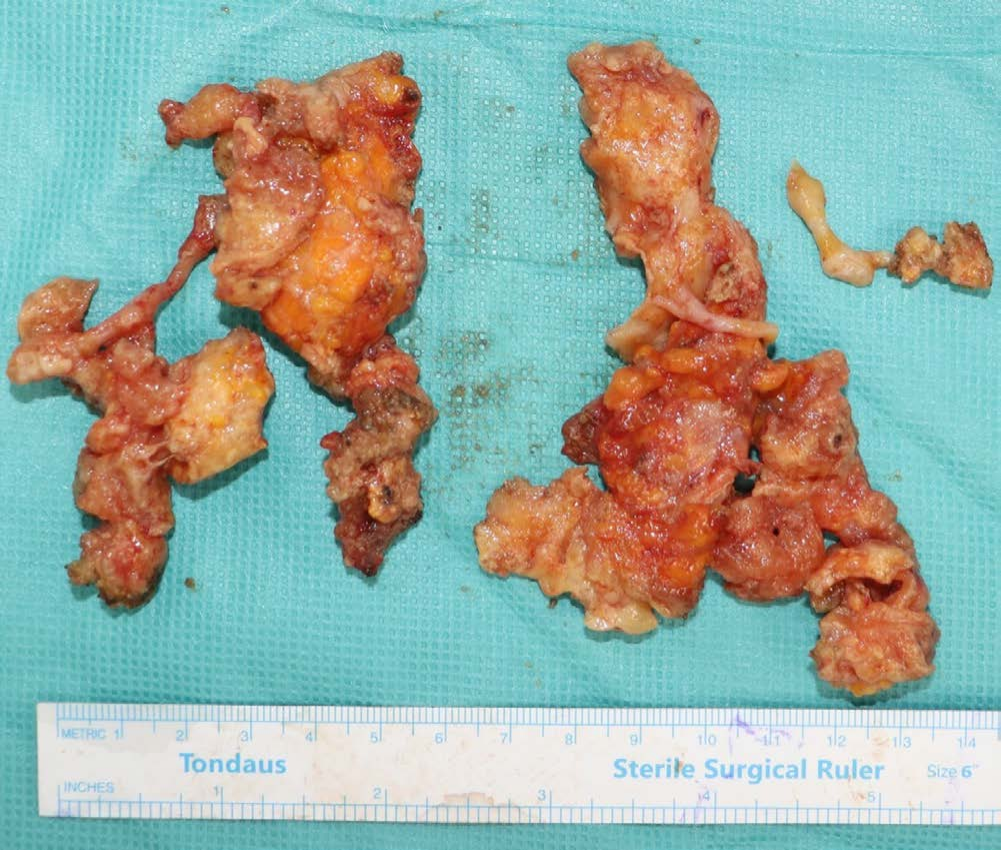
Intraoperative photography. The granuloma was removed from both breasts.

**Figure 5.**
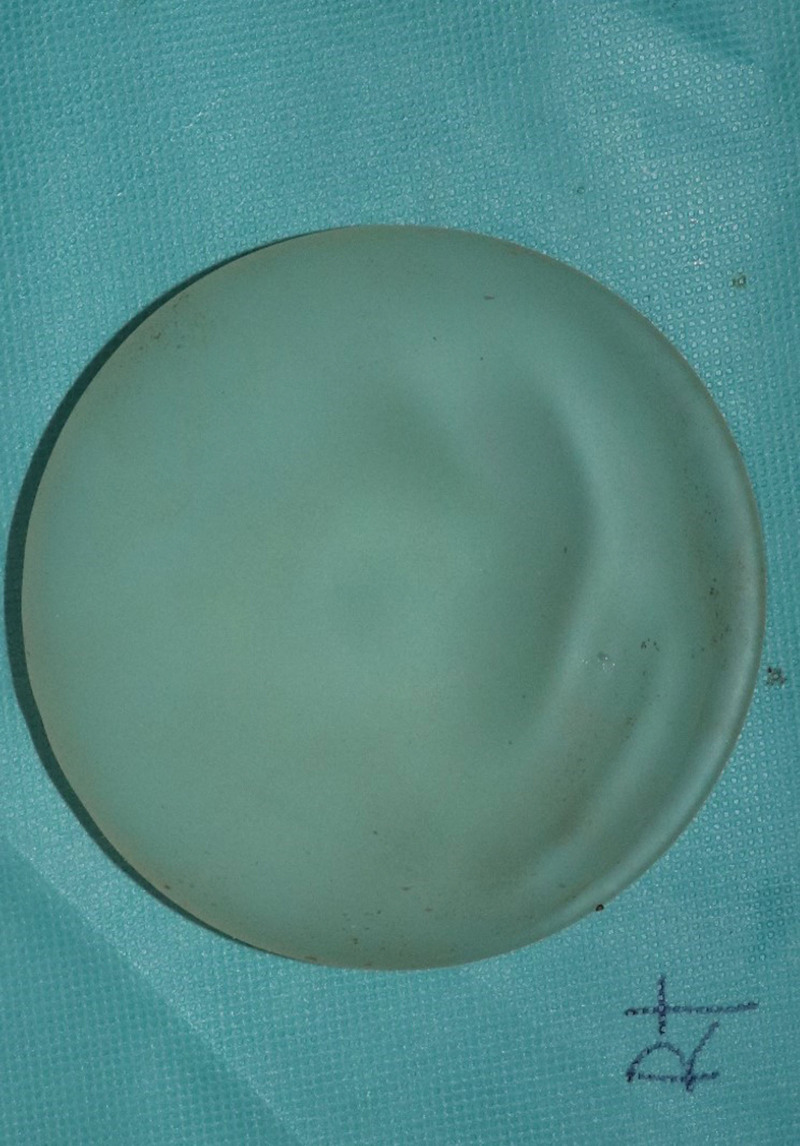
Intraoperative photography. The implant was micro-textured implant, and removed.

**Figure 6.**
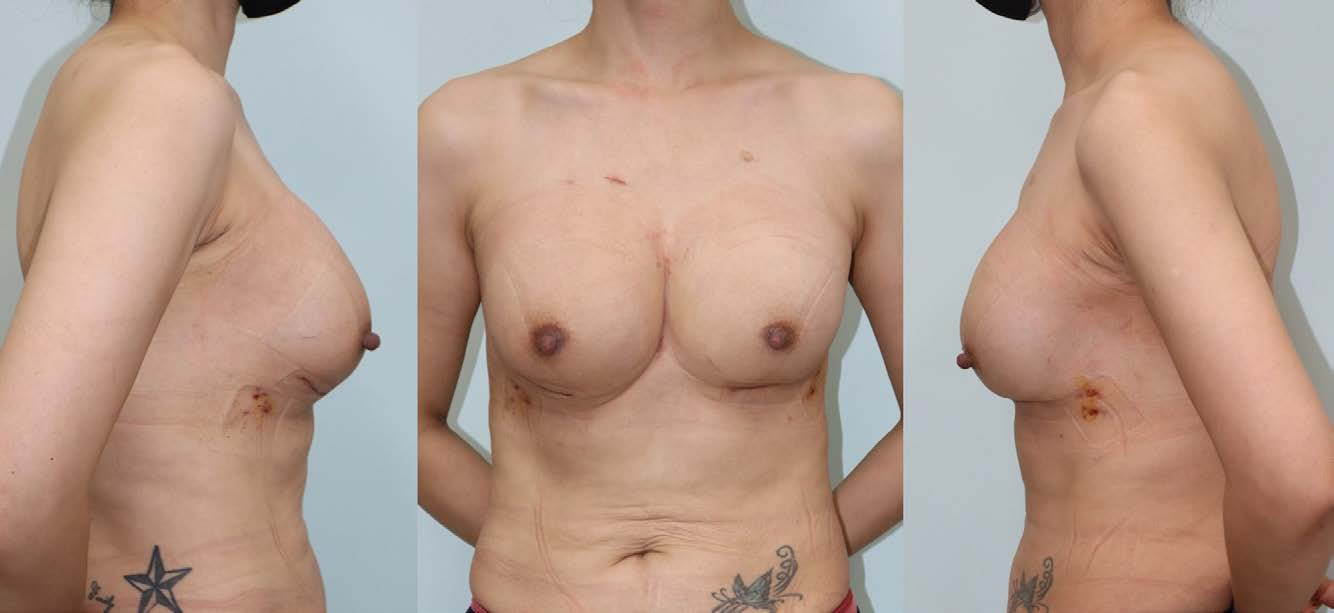
Immediate postoperative photography on postoperative day 10th.

Implant removal and capsulectomy of both breasts were performed under general anesthesia. The patient opted to replace the new implant with a smaller one, immediately after the removal of the old implant. To remove the capsule easily, incision was done through the inframammary fold approach. In the intraoperative field before implant exposure, multiple fat necrotic lesions were observed, with massive fluid collection of more than 200 mL in the prepectoral plane (Fig. 3). Fat necrosis and an unspecified granuloma with severe adhesions to the glandular tissue, pectoralis major muscle, and breast parenchyma were observed. Therefore, it was presumed that the unspecified granuloma may be due to an alloplastic filler, which was not initially reported by the patient. The fluid, fat necrosis, and foreign bodies around the granuloma were removed as much as possible, and massive irrigation was performed (Fig. 4). After incision of the pectoralis major muscle, the previously inserted implant was observed (Fig. 5), which migrated severely to the upper lateral portion of the breast. Migration of both implants to the axilla causes inadequate breast projection. However, regardless of implant malposition, the capsule was clean and lesions suspicious for contracture were not observed. We created a new pocket with dissection in the inferomedial direction and sutured the previous capsule. Massive irrigation was performed with povidone-iodine and saline solution inside the pocket, and a 175-cc smooth implant (Memory Gel, Mentor, California) was inserted using the dual plane method (Fig. 6).

**Figure 7.**
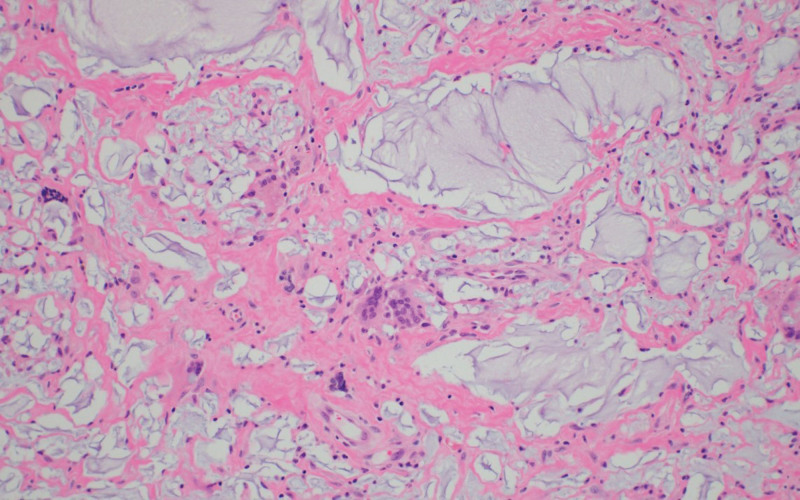
Pathologic finding. Amorphous material and foreign body giant cells are observed (H&E, ×100).

There were no findings suggestive of malignancy on cell cytology, such as CD30 negativity. Biopsy of the implant capsule and granuloma confirmed a foreign body reaction infiltrating the tissue with inflammation. The lesion shows diffuse infiltration of amorphous material with foreign body giant cells (Fig. 7). No bacterial growth was observed in the tissue cultures.

**Figure 8.**
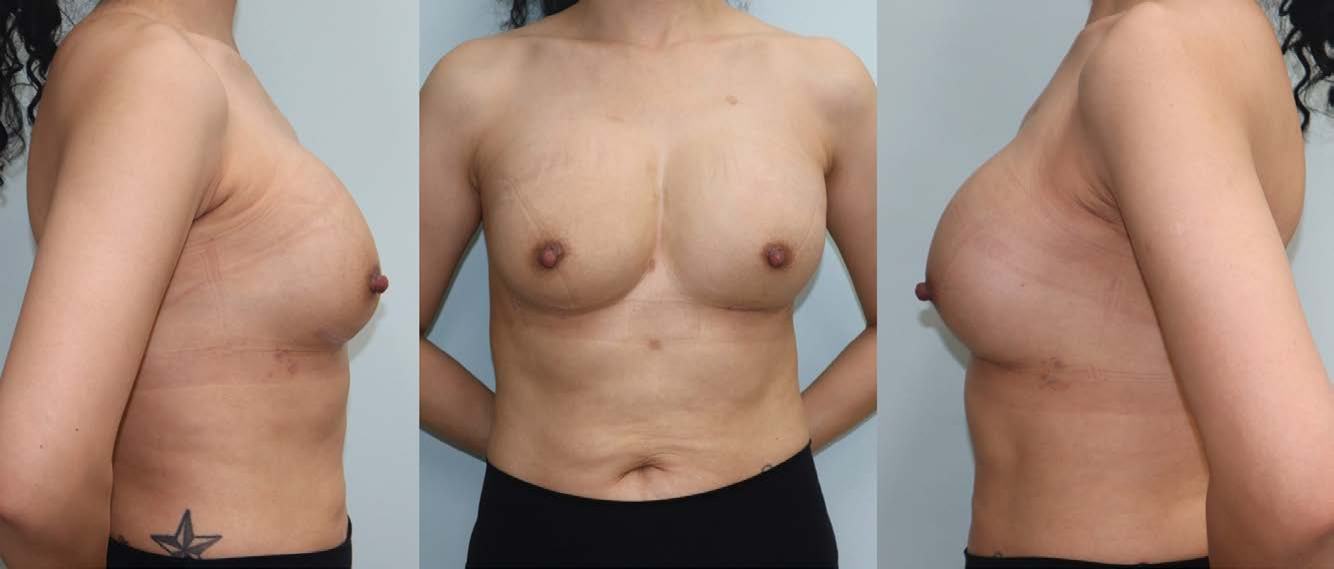
Postoperative photography on 4 mo follow-up.

After the surgery, the patient stated that the result of the previous breast augmentation was unsatisfactory; therefore, she underwent an additional procedure using an unspecified alloplastic filler at the same local clinic 3 months after the initial surgery. The amount and name of the injected filler was undocumented, but the patient reported that it was not an autologous fat grafting. After the immediate implant change, excessive protrusion of both breasts was corrected without any filler, and complications such as hematoma, seroma, capsular contracture, and inflammation were not observed at the 4-month follow-up (Fig. 8). In summary, the patient had granuloma and a large amount of fluid collection due to an unspecified filler injection by a non-specialist; thus, implant migration was concealed and misconceived as implant rupture.

## 4. Discussion

The types of fillers that can be injected directly include autologous fats and alloplastic fillers, such as silicone, paraffin, and recently developed hyaluronic acid (HA) and polyacrylamide hydrogel (PAAG). This procedure is convenient because filler injections can be performed under local anesthesia.^[2]^ Autologous fat grafting is widely used to fill insufficient volumes after breast implant augmentation, since there is no risk for foreign body reaction. However, its disadvantages include fat necrosis and calcification, which are limited because it cannot produce dramatic results due to reabsorption.^[6,7]^

HA is a glycosaminoglycan found naturally in the body and has a short half-life. In addition, as it is a substance that is absorbed into the body, it is generally safe. However, because breast augmentation requires a comparatively larger volume of filler, it is very expensive, and its nonpermanent characteristics are problematic. Macrolane, a well-known derivative of HA, is the newest product available in the breast augmentation filler market. Launched in the United Kingdom market in 2008, it was promoted for use in the volume restoration of soft tissues, including the breasts. It is a gel component composed of HA that is injected deep into the soft tissue and is gradually absorbed into the body over 12 months. It was considered relatively safe, but some cases of infection and capsular contracture have been reported after injection of Macrolane.^[8]^

Alloplastic fillers can cause various complications owing to foreign body reactions. Granulomas, infections, and filler migration have been reported in cases of PAAG implantation.^[9,10]^ PAAG filler (Amazingel, NanFeng Medical Science and Technology Development Co., Ltd., Shiji azhuang, China) was previously widely used in China, but it was banned in 2006 due to related complications.^[11]^ PAAG gel materials may have toxicity, and concerns regarding tumor formation remain.^[12,13]^

In the 2010s, AQUAfilling (Biomedica, Prague, Czech Republic) was introduced to overcome the limitations of existing fillers. In order to create a natural and satisfactory size and shape after augmentation surgery using implants, it was suggested to inject AQUAfilling for body contouring into the space between the implant and the skin. It has since been widely used in Turkey, Germany, Malaysia, Japan, and Korea.^[14]^ It is a hydrophilic gel composed of 98% sodium chloride solution (0.9%) and 2% cationic polyamide and is biocompatible with human tissue.^[5]^ It was approved as a dermal filler by the Korean Food and Drug Administration in 2013. It was initially used as a skin filler for the face and buttocks; however, its use as a body filler has expanded, and it has become a popular choice for breast augmentation.^[5]^ Although injection is easy and has long-term clinical effects, many cases of complications such as infection and toxicity due to penetration into the surrounding tissue have been reported.^[14,15,16]^

In our case, the injected filler was unknown. However, the injection of AQUAfilling was the most suspicious because many complications of AQUAfilling have been reported recently and the patient underwent the procedure in Korea. It was not absorbed for 4 years, and the shape of the breast was maintained; therefore, it may be a non-absorbable alloplastic filler. Based on the fact that AQUAfilling has been widely used in patients in Korea recently, it could be the most suspicious for this case. However, because the exact composition of the filler was unknown, it was necessary to determine whether the fluid collection on MRI was due to implant-related complications or inflammation caused by the filler.

Radiographic imaging provides important information for diagnosing patients who develop complications after breast augmentation with implants or filler injections. Radiographic imaging methods include mammography, sonography, and MRI. Among these, MRI has the advantage of accurately imaging the extent of fluid collection caused by the implant or filler as well as mass-like lesions around the implant.^[17]^ In our case, extracapsular fluid collection was observed on MRI. In general, in the early stages after implant insertion, the immune system against a foreign body produces a capsule around the implant, and a small amount of fluid collection may normally occur.^[18]^ However, if capsular inflammation and fluid accumulation accompany a prosthetic infection, prompt implant removal is required. Even in BIA-ALCL, fluid collection around the implant has been observed in most cases. Typically, malignant fluid collection occurs 2 years after implant insertion.^[19]^ As the patient in our case visited the hospital 4 years after the implant was inserted without knowing exactly which filler was used, it was necessary to consider and investigate for malignancy.

Recently, a new radiographic finding of silicone-induced granuloma of the breast implant capsule (SIGBIC) was reported as a complication associated with silicone implants.^[20]^ SIGBIC is defined as a granuloma produced by an immune reaction between the capsule of the implant and free silicone, and is presumed to be caused by gel bleeding without any rupture of the implant.^[21]^ Similar to BIA-ALCL, SIGBIC is often observed in the form of a large volume of intracapsular fluid collection and an intracapsular mass; therefore, differential diagnosis is important. Because SIGBIC is difficult to differentiate from BIA-ALCL, if an intracapsular mass and fluid collection are suspected on radiological imaging, a biopsy or cell cytology should be performed. SIGBIC is histologically characterized by clustered lymphocytes surrounded by a fibrous capsule and extracapsular silicone.^[22]^ In our case, a foreign body reaction due to gel bleeding also needed to be considered because there was no implant rupture in the intraoperative field. However, considering the clean capsule and absence of intracapsular fluid collection, the possibility of SIGBIC seemed low. Consequently, the filler injection history was identified; therefore, the complication may be more attributable to the filler.

Generally, it is not safe to perform reconstruction with silicone implants immediately after the removal of alloplastic fillers. This is because the coexistence of the new implant and remaining filler hinders the stability of the new implant. However, some researchers recommend immediate reconstruction after filler removal because it reduces patient stress and avoids the discomfort associated with secondary surgery.^[23]^ In the case of filler complications, some patients prefer reconstructive surgery after the removal of the filler. Based on this, Choi et al proposed a diagnostic algorithm.^[24]^ MRI is recommended as the primary diagnostic tool in this algorithm, and immediate reconstruction is possible if the preexisting capsule is healthy; however, it is based on careful judgment of the surgeon. In our patient case, there were no abnormal laboratory findings, and a healthy capsule was identified; therefore, we immediately performed reconstruction using silicone implants. There was no recurrence of infection 4 months after the surgery. However, further studies are required to confirm the safety and complications of immediate implant placement after filler removal.

We report a case in which a filler granuloma due to a large amount of filler injection was mistaken for breast implant rupture. Complications such as infection and granuloma due to alloplastic filler injection have been reported in Korea and have become increasingly problematic in recent years. However, in most reported cases, complications occur only after filler injection. Complications are relatively rare in patients who have undergone both implant augmentation and filler injections. Even in the literature, there is only 1 case of implant rupture 1 year after AQUAfilling injection.^[25]^ In this case, it was not clear whether the cause of the implant rupture was the implant itself or AQUAfilling. Complications related to breast fillers have been increasing recently, but they do not show consistent symptoms, and it is not easy to predict aesthetic outcomes. Therefore, surgeons should make a sufficient surgical plan to explain the limitations of postoperative aesthetic outcomes to patients. In particular, in patients who undergo both implant augmentation and filler injection, it is much more difficult to determine the cause of complications and predict surgical outcomes. Accurate history taking and establishment of a surgical plan with sufficient radiographic imaging are necessary to achieve better surgical results and minimize postoperative complications.

## 5. Conclusions

Injection of a large amount of breast filler can eventually cause symptoms such as granuloma due to infiltration into the surrounding tissue and foreign body reactions, resulting in swelling, pain, tenderness, and breast asymmetry. It was necessary to determine whether the fluid collection on MRI was caused by implants or fillers. We report a case in which satisfactory results were obtained without any complications by performing immediate breast reconstruction using silicone implants after removing the infective alloplastic fillers. Surgeons should be aware of complications associated with alloplastic filler injections. In addition, if a large amount of fluid is collected by the breast filler, malpositioning of the implant can occur, and in severe cases, it can be mistaken for rupture, as in our case.

## Author contributions

**Conceptualization:** Da Woon Lee.

Writing – original draft: Yong Seon Hwang.

Writing – review & editing: Je Yeon Byeon, Jun Hyuk Kim, Hwan Jun Choi, Mee Hye Oh, Da Woon Lee.
